# Autophagy Suppresses Toll-Like Receptor 3-Mediated Inflammatory Reaction in Human Epidermal Keratinocytes

**DOI:** 10.1155/2020/4584626

**Published:** 2020-05-01

**Authors:** Xue Mei Li, Kyung Eun Jung, Su Hyuk Yim, Dong Kyun Hong, Chang Deok Kim, Jeong Yeon Hong, Ho Jung Lee, Sung Yul Lee, Jung Eun Kim, Chang Wook Park

**Affiliations:** ^1^Department of Dermatology, School of Medicine, Chungnam National University Hospital, 282 Munhwa-ro, Jung-gu, Daejeon 35015, Republic of Korea; ^2^Department of Medical Science, School of Medicine, Chungnam National University, 99 Daehak-ro, Yuseong-gu, Daejeon 34134, Republic of Korea; ^3^Department of Dermatology, Soonchunhyang University Cheonan Hospital, Soonchunhyang University College of Medicine, 31, Suncheonhyang 6-gil, Dongnam-gu, Cheonan 31151, Republic of Korea; ^4^Department of Medicine, Soonchunhyang University College of Medicine, 22, Soonchunhyang-ro, Sinchang-myeon, Asan 31538, Republic of Korea

## Abstract

Autophagy, one mechanism of programmed cell death, is fundamental to cellular homeostasis. Previous studies have identified autophagy as a novel mechanism by which cytokines control the immune response. However, its precise role in immune-related inflammatory skin diseases such as psoriasis remains unclear. Thus, this study explored the functional role of autophagy in psoriatic inflammation of epidermal keratinocytes. Strong light chain 3 immunoreactivity was observed in epidermal keratinocytes of both human psoriatic lesions and imiquimod-induced mice psoriatic model, and it was readily induced by polycytidylic acid (poly (I:C)), which stimulates Toll-like receptor 3 (TLR3), in human epidermal keratinocytes in vitro. Rapamycin-induced activation of autophagy significantly reduced poly (I:C)-induced inflammatory reaction, whereas, inhibition of autophagy by 3-methyladeine increased that. Our results indicate that the induction of autophagy may attenuate TLR3-mediated immune responses in human epidermal keratinocytes, thus providing novel insights into the mechanisms underlying the development of inflammatory skin diseases including psoriasis.

## 1. Introduction

Psoriasis is a chronic immune-mediated inflammatory skin disease characterized by distinct erythematous plaques with silvery scales, affecting approximately 2 to 3% of the population worldwide [1, 2]. However, the molecular mechanisms involved in the pathogenesis of psoriasis are not clear to date. Numerous studies have shown that activated T lymphocytes are required for the development and persistence of immune responses in psoriatic skin, including Th17 cell-derived cytokines such as interleukin- (IL-) 17A. Nevertheless, psoriasis cannot be considered uniquely as a T cell-dependent disease [3], as dysregulated cross talk between immune cells and skin-resident keratinocytes is thought to be essential in psoriasis development [4–6]. Recent data suggest that keratinocytes also play a pivotal role in triggering early pathogenic events, as well as in sustaining the chronic plaque of psoriasis [7–9]. Skin-resident keratinocytes not only act as inducers of innate immune responses in the early phase, they are also involved in adaptive immune responses and serve as a reservoir of inflammatory mediators necessary for sustained psoriatic lesions [3].

Autophagy is a process of cellular recycling and degradation that is highly conserved in all eukaryotes [10]. Associations between autophagy and various human disorders have been known since the 1950s [11, 12]. While previous research has identified an association between autophagy and autoimmune skin disorders, the precise mechanism remains unknown [13]. Recent studies have also demonstrated an association between autophagy and innate immune responses [14]. Given that keratinocytes and its immune responses plays an essential role in the pathogenesis of psoriasis, we postulated that autophagy may be involved in psoriatic inflammation of keratinocytes. Here, we investigated whether autophagy is correlated with psoriatic inflammation of epidermal keratinocytes. A better understanding of the effect of autophagy associated with polycytidylic acid- (poly (I:C)-) induced Toll-like receptor 3- (TLR-3-) mediated inflammation can contribute to research of new possible treatment options for immune-related inflammatory skin disorders.

## 2. Materials and Methods

### 2.1. Immunohistochemistry (IHC)

Tissue samples were fixed with 10% formaldehyde, embedded in paraffin, and cut into 4 *μ*m thick sections. Sections were deparaffinized in xylene and then rehydrated using an alcohol series. For IHC, sections were first treated with 3% H_2_O_2_ to block the endogenous peroxidase and then incubated with IHC protein block solution (DAKO, Carpinteria, CA, USA). The sections were then reacted with light chain 3 (LC3) antibody (Sigma-Aldrich, St. Louis, MO, USA) at 4°C for overnight, followed by horseradish peroxidase-conjugated secondary antibodies (DAKO). After washing, the sections were incubated with diaminobenzidine tetrachloride solution and counterstained with Mayer's hematoxylin.

### 2.2. Animal Models

An imiquimod-induced psoriasis model was established as described previously [15]. Seven-week-old male BALB/c mice were obtained from Orient Bio (Seongnam, Korea). After the removal of all dorsal hair using electric clippers, 5% imiquimod cream (Aldara Cream, Dong-A ST, Seoul, Korea) was topically applied for 7 days. Skin specimens were prepared at the indicated time points and analyzed by IHC.

### 2.3. Cell Culture

Human skin tissues were obtained under the written informed consent of the donors. All procedure were approved by the Institutional Review Board (IRB) of Chungnam National University Hospital (IRB No.1011-135). Primary keratinocytes were isolated from the epidermis and then immortalized using a recombinant retrovirus expressing simian virus 40 T antigen [15]. Immortalized human epidermal keratinocytes were routinely cultured in keratinocyte-serum free medium supplemented with bovine pituitary extract and recombinant human epidermal growth factor (Life Technologies Corporation, Grand Island, NY, USA).

### 2.4. Monitoring Autophagy

cDNA for LC3B was obtained by reverse transcription-polymerase chain reaction (RT-PCR). Briefly, total RNA was isolated from keratinocytes using an easy-BLUE RNA extraction kit (Intron, Daejeon, Korea), after which 2 *μ*g of total RNA was reverse-transcribed with the Moloney-murine leukemia virus (M-MLV) reverse transcriptase (Elpis Biotech, Daejeon, Korea). An aliquot of RT mixture was then subjected to PCR amplification using a primer set for LC3B (5′-GTACGGATCCATGCCGTCGGAGAAGACCTT and 5′-AATTCTCGAGTTACACTGACAATTTCATCC). The amplified full-length cDNA for LC3B was then subcloned into the pENT/cytomegalovirus-green fluorescent protein (GFP), and replication-incompetent adenoviruses were created as reported previously [16]. For monitoring autophagy, the cells were transduced with adenovirus expressing GFP-LC3B overnight, after which the cells were replenished with fresh growth medium and incubated for an additional 24 h. After treatment with poly (I:C), autophagy was monitored by observation of LC3 puncta under the fluorescent microscopy.

### 2.5. Western Blot Analysis

Cells were lysed in PRO-PREP solution (Intron), with the total protein measured using a bicinchoninic acid assay protein assay kit (Thermo Scientific, Rockford, IL, USA). Samples were loaded onto SDS-polyacrylamide gels and transferred to nitrocellulose membranes (Pall Crop, East Hills, NY, USA). After blocking with 5% skim milk, the membranes were incubated with primary antibodies. The blots were then incubated with peroxidase-conjugated secondary antibodies and visualized by enhanced chemiluminescence (Intron). The following primary antibodies were used: LC3 (Sigma-Aldrich); nucleotide-binding oligomerization domain-like receptor protein 3 (NLRP3) and apoptosis-associated speck-like protein containing a caspase recruitment domain (ASC; AdipoGen, San Diego, CA, USA); IL-1*β* (Abcam, Cambridge, MA, USA); caspase-1 (Cell Signaling Technology, Beverly, MA, USA); and actin (Santa Cruz Biotechnology, Santa Cruz, CA, USA).

### 2.6. Quantitative Real-Time Polymerase Chain Reaction

For quantitative real-time polymerase chain reaction (qRT-PCR), 2 *μ*g of total RNA was reverse-transcribed with M-MLV reverse transcriptase, as described above. Equal volumes of cDNA were then used as templates for PCR using the SYBR Green Master Mix (Elpis Biotech, Daejeon, Korea). qRT-PCR was performed using the ABI StepOne Real-Time PCR system (Applied Biosystems, Foster City, CA, USA). The gene-specific primers used were as follows: IL-8, 5′-CCTTTCCACCCCAAATTTATCA and 5′-TTTCTGTGTTGGCGCAGTGT; IL-6, 5′-CTGCGCAGCTTTAAGGAGTTC and 5′-CCATGCTACATTTGCCGAAGA; IL-1*β*, 5′-TTAAAGCCCGCCTGACAGA and 5′-GCGAATGACAGAGGGTTTCTTAG; tumor necrosis factor-*α* (TNF-*α*), 5′-CTCCTTCAGACACCCTCAACCT and 5′-CGACCCTAAGCCCCCAATT; C-C motif chemokine 20 (CCL20), 5′-CCACCTCTGCGGCGAAT and 5′-TGTGTATCCAAGACAGCAGTCAAA; and GAPDH, 5′-TGCACCACCAACTGCTTAGC and 5′-GGCATGGACTGTGGTCATGAG.

### 2.7. Enzyme-Linked Immunosorbent Assay

Culture medium was collected, and the protein levels of secreted IL-8, IL-6, and IL-1*β* were determined using commercial enzyme-linked immunosorbent assay (ELISA) kits. The IL-8 kit was purchased from Thermo Scientific; IL-6 and IL-1*β* kits were purchased from R&D Systems (Minneapolis, MN, USA).

### 2.8. Statistical Analysis

Statistical analyses were performed by a one-way ANOVA or Student's *t*-test, as appropriate, using the SPSS software version 22.0 (IBM Corp., Armonk, NY, USA). A *p* value of less than 0.05 was considered significant.

## 3. Results

### 3.1. LC3, An Autophogosome Marker, Is Highly Expressed in Psoriatic Skin

**Figure 1:**
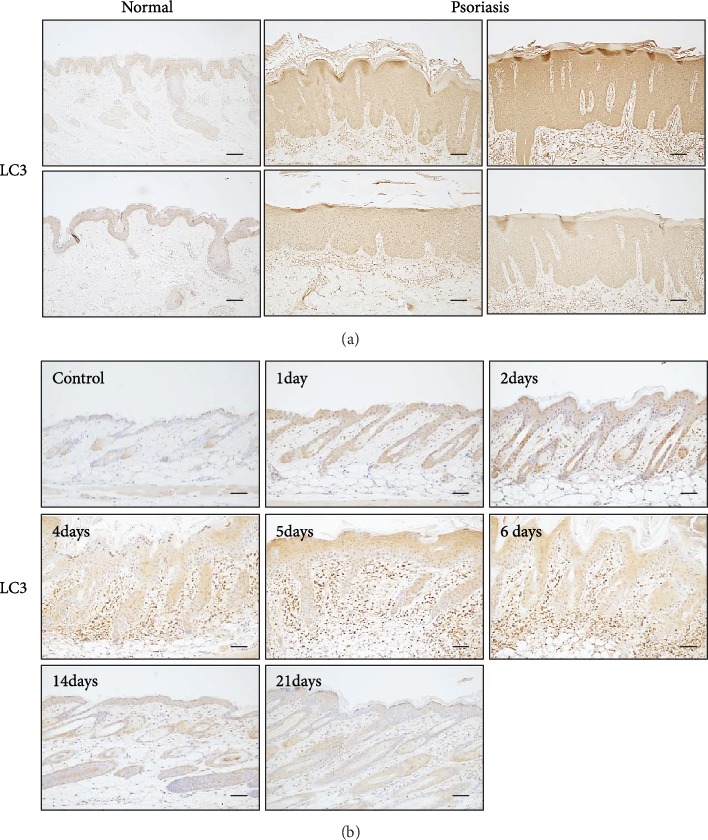
(a) Skin specimens were obtained from normal and psoriatic lesions, and immunohistochemistry was performed using the anti-light chain 3 (LC3) antibody. The LC3 level in psoriatic lesions was higher than that of normal epidermis. (b) BALB/c mice were topically applied with imiquimod cream. The LC3 level in imiquimod-treated epidermis was markedly increased compared to control epidermis.

To investigate the relationship between autophagy and psoriasis, we first performed IHC against the autophagy marker LC3 using skin specimens obtained from normal and psoriatic lesions. LC3 was weakly detected in normal epidermis, while strong LC3 immunoreactivity was observed in epidermal keratinocytes in psoriatic lesions (Figure 1(a)). To further explore the possible involvement of autophagy in psoriasis, we used a well-established imiquimod-induced psoriasiform model [15]. When the imiquimod cream was applied topically to the mouse's back skin, the epidermal thickness was markedly increased in a time-dependent manner. Similar to the IHC results from psoriatic lesions, LC3 immunoreactivity was strongly detected in thickened epidermis. The LC3 levels began to increase within 24 h after treatment with imiquimod and lasted for up to 7 days (Figure 1(b)). These results suggest that autophagy play a role in psoriasis.

### 3.2. Effects of Poly (I:C) on Autophagy in Keratinocytes

**Figure 2:**
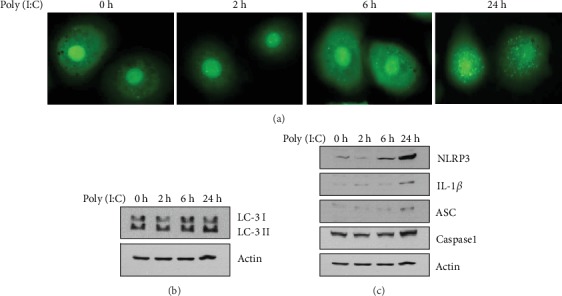
(a) Keratinocytes were transduced with recombinant adenovirus expressing green fluorescent protein-light chain 3 (GFP-LC3) overnight. After changing the medium, the cells were further incubated with fresh growth medium for 1 day. The cells were then treated with 1 *μ*g/mL of poly (I:C) for the indicated time points. Autophagic LC3 puncta were observed under fluorescent microscopy. Autophagy was induced by poly (I:C) in a time-dependent manner. (b) Keratinocytes were treated with poly (I:C) for the indicated time points. The LC3 level was determined by western blot. Poly (I:C) increased the LC3-II level, indicating that autophagy was induced. (c) Effect of poly (I:C) on inflammasome-related molecules. Poly (I:C) increased the protein level of nucleotide-binding oligomerization domain-like receptor protein 3 (NLRP3), interleukin-1-beta (IL-1*β*), apoptosis-associated speck-like protein containing a caspase recruitment domain (ASC), and caspase 1.

Epidermal keratinocytes express TLR3, and stimulation of TLR3 induces innate immune-related inflammatory reaction in epidermal keratinocytes [17]. Poly (I:C) is an analogue of double-stranded RNA (dsRNA), which induces inflammatory reaction via TLR3-activaion, and it has been described that psoriatic keratinocytes exhibit increased sensitivity to viral RNA intermediates [18]. Thus, poly (I:C) is widely used for studying innate immunity-related skin diseases such as psoriasis [19]. To investigate the effects of poly (I:C) on autophagy, we transduced keratinocytes with a recombinant adenovirus expressing GFP-LC3 and then treated them with poly (I:C). Treatment with poly (I:C) was shown to activate autophagy as evidenced by increased LC3 puncta (Figure 2(a)). Consistent with these data, poly (I:C) also increased the protein levels of LC3-II, which is reflective of the number of autophagosomes and autophagy-related structures (Figure 2(b)) [19]. Parallel to these experiments, we examined the effect of poly (I:C) on the levels of several inflammasome-related proteins. Treatment with poly (I:C) was shown to induce a variety of inflammasome-related molecules including NLRP3, IL-1*β*, ASC, and caspase 1 (Figure 2(c)). These data suggest that autophagy plays a role in the inflammatory reaction of epidermal keratinocytes.

### 3.3. Suppressive Role of Autophagy in Poly (I:C)-Induced Inflammatory Reaction

**Figure 3:**
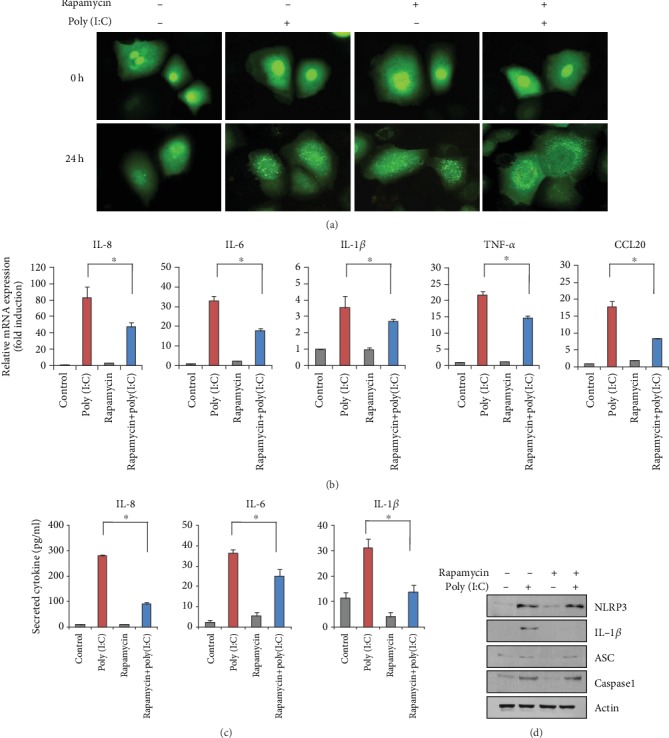
(a) Keratinocytes were transduced with recombinant adenovirus expressing green fluorescent protein-light chain 3 (GFP-LC3) overnight. After changing the medium, the cells were further incubated with fresh growth medium for 1 day. The cells were then treated with 1 *μ*g/mL of poly (I:C) and rapamycin (1 *μ*M). Autophagic LC3 puncta were observed under fluorescent microscopy. Cotreatment with rapamycin enhanced autophagy induction. (b) Cells were cotreated with poly (I:C) and rapamycin for 2 h. The messenger RNA (mRNA) level for inflammatory cytokines were determined by real-time polymerase chain reaction. Rapamycin cotreatment inhibited poly (I:C)-induced gene expression of inflammatory cytokines. (c) The cells were cotreated with poly (I:C) and rapamycin for 24 h. The conditioned media were collected, and secreted inflammatory cytokines were determined by enzyme-linked immunosorbent assay. Rapamycin cotreatment inhibited poly (I:C)-induced secretion of inflammatory cytokines. (d) Effect of rapamycin on inflammasome-related molecules.

**Figure 4:**
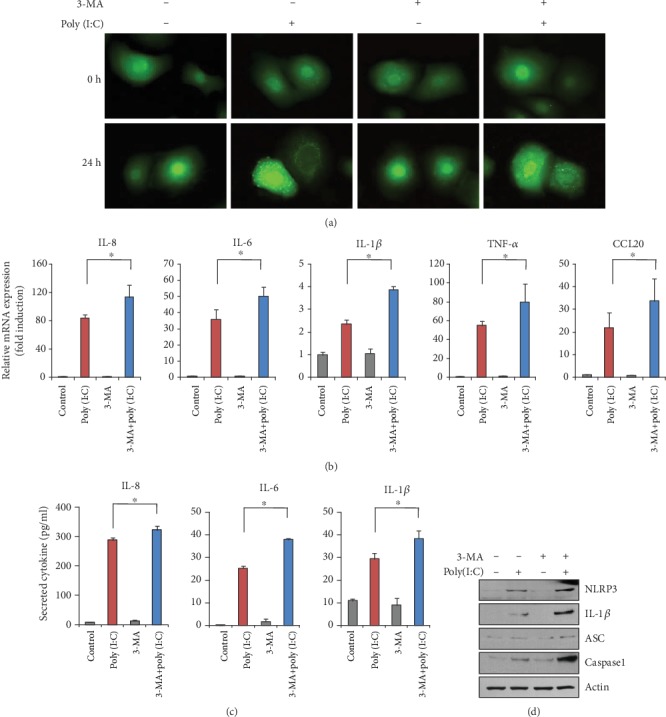
(a) Keratinocytes were transduced with recombinant adenovirus expressing green fluorescent protein-light chain 3 (GFP-LC3) overnight. After changing the medium, the cells were further incubated with fresh growth medium for 1 day. The cells were then treated with 1 *μ*g/mL of poly (I:C) and 3-methyladenine (3-MA) (5 *μ*M). Autophagic LC3 puncta were observed under fluorescent microscopy. Cotreatment with 3-MA inhibited poly (I:C)-induced autophagy. (b) The cells were cotreated with poly (I:C) and 3-MA for 2 h. The messenger RNA (mRNA) level for inflammatory cytokines was determined by real-time polymerase chain reaction. The 3-MA cotreatment enhanced poly (I:C)-induced gene expression of inflammatory cytokines. (c) The cells were cotreated with poly (I:C) and 3-MA for 24 h. The conditioned media were collected, and secreted inflammatory cytokines were determined by enzyme-linked immunosorbent assay. The 3-MA cotreatment enhanced poly (I:C)-induced secretion of inflammatory cytokines. (d) Effect of 3-MA on inflammasome-related molecules.

To examine the role of autophagy in poly (I:C)-induced inflammatory reaction, we cotreated keratinocytes with poly (I:C) and rapamycin, a potent mechanistic target of rapamycin complex1 inhibitor and well-known inducer of autophagy. Cotreatment with poly (I:C) and rapamycin significantly increased LC3 puncta compared to poly (I:C)- and rapamycin-only treated groups, indicating that autophagy was enhanced (Figure 3(a)). We then examined the effects of these treatments on the expression of inflammatory cytokines. Poly (I:C) markedly induced the mRNA levels of several inflammatory cytokines, including IL-8, IL-6, IL-1*β*, TNF-*α*, and CCL20. These effects were significantly attenuated in cells cotreated with rapamycin (Figure 3(b)). Similarly, the poly (I:C)-induced secretion of inflammatory cytokines was significantly reduced by rapamycin (Figure 3(c)). Consistent with these data, rapamycin slightly reduced the expression of inflammasome-related molecules (Figure 3(d)). Next, we cotreated keratinocytes with poly (I:C) and 3-methyladeine (3-MA), an agent that blocks the formation of autophagosomes. Cotreatment with 3-MA significantly decreased LC3 puncta formation, indicating that poly (I:C)-induced autophagy was effectively inhibited (Figure 4(a)). Inhibition of poly (I:C)-induced autophagy resulted in a significant increase in inflammatory gene expression and the secretion of inflammatory cytokines (Figures 4(b) and (c)). Similarly, cotreatment with 3-MA resulted in the significant induction of inflammasome-related molecules (Figure 4(d)). Collectively, these data suggest that autophagy exerts a suppressive effect on inflammatory gene expression in keratinocytes.

## 4. Discussion

The aim of our study was to characterize the relationship between autophagy and innate immune-related inflammatory reaction in epidermal keratinocytes. Psoriasis is a chronic, immune-mediated inflammatory skin disorder, which arises in genetically susceptible individuals who have an abnormal immune response to environmental factors. There is emerging evidence for the role of psoriatic keratinocytes in terms of immune-regulatory functions. Keratinocytes, the main constituents in the epidermis, function as an essential mechanical as well as immunologic barrier against pathogens and are important producers of proinflammatory cytokines and chemokines such as IL-1*β*, tumor necrosis factor-*α*, IL-6, IL-8, and CCL20 [8]. Inflammatory cytokines secreted by psoriatic keratinocytes are known to play important roles in the recruitment and activation of neutrophils and T cells, leading to excessive proinflammatory responses and maintenance of inflammation [9, 20]. Keratinocytes detect the danger signals via TLR system and initiate innate immune responses. Among various functional TLRs expressed by keratinocytes, TLR3 recognizes dsRNA released from some viruses. Psoriatic keratinocytes are susceptible to viral RNA intermediates, and activation of TLR3 leads to increase of proinflammatory cytokines related with psoriasis. Poly (I:C) has been used extensively as an immune-stimulant that activates TLR3 ligand. Thus, we used poly (I:C) to evaluate the innate immune-related inflammatory reaction, in this study.

Innate immune response has been shown to induce autophagy under various conditions. In this study, activation of innate immune response via treatment with poly (I:C) led to increased expression of autophagy markers and the formation of autophagosomes, along with decreased inflammatory cytokine expression in the induced autophagy group, relative to the suppressed autophagy group. Based on these results, we hypothesized that autophagy suppresses poly (I:C)-induced, TLR3-mediated keratinocyte inflammation. Autophagy refers to a cellular catabolic process of conveying intracellular or extracellular contents to lysosomes for their degradation [21]. Autophagy has a critical role in intracellular homeostasis by eliminating senescent or damaged organelles and accumulated cellular contents [22]. Under stressful situations, such as hypoxia, oxidative stress, radiation, or infection, autophagy is activated to sustain cellular survival [21]. Given the importance of autophagy to the maintenance and survival of cells, dysregulated autophagy has been thought to be linked to a variety of human disorders, including neurodegeneration, cancer, myopathies, aging, infections, and inflammatory diseases [21, 23]. Numerous dermatologic diseases, including psoriasis, vitiligo, systemic lupus erythematosus, systemic sclerosis, cancer, and infectious diseases, have been shown to exhibit various levels of autophagic dysregulation [24, 25]. So far, the role of autophagy in these conditions has been interpreted based on the association of autophagy with inflammation, keratinocyte differentiation, and melanocyte survival [24, 26]. Recent studies have suggested that autophagy plays an important role in cell apoptosis, antigen presentation, pathogen clearance, and inflammation [27]. Regulation of these inflammatory responses can be explained either directly as a result of autophagy acting on stability or secretion of inflammatory mediators or indirectly by suppressing intracellular stressors [28]. Other recent studies suggest that autophagy plays an important role in the regulation of inflammation by disrupting inflammasome activation [29]. Another crucial anti-inflammatory role of autophagy is to negatively regulate type I interferon production [30]. Thus, autophagy dysfunction may mediate susceptibility to inflammatory skin diseases including psoriasis.

Psoriasis is a chronic inflammatory skin disease of unknown etiology, with significant questions remaining in regard to the mechanisms underlying the disease. Recently, the pathogenesis of psoriasis is explained by the dysregulation of immune cell function and keratinocyte differentiation [31]. Interestingly, defects in autophagy have been linked to both pathological characteristics [27]. Moreover, a recent study revealed that several single nucleotide polymorphisms in the ATG16L1 gene are linked with susceptibility to the development of psoriasis [32]. However, despite considerable research on autophagy, the exact mechanism underlying autophagy-mediated pathology in chronic inflammatory skin disease, such as psoriasis, remains unknown. A previous study showed that autophagy negatively downregulates keratinocyte inflammatory responses [33]. Hence, we postulated that autophagy dysregulation contributes to the development of psoriasis via one or more immune mechanisms and we sought to better understand the role of autophagy in the pathogenesis of psoriasis. The results of this study provide insight into the role of autophagy in innate immune-mediated psoriatic inflammation in epidermal keratinocytes. Our experiments show that inflammatory cytokine production is mediated by a disruption in keratinocyte autophagy. These observations are consistent with previous reports showing that autophagy is able to modulate cytokine production at the posttranslational level by degrading components of the inflammasome, including the molecular complex that cleaves pro-IL-1*β* into its bioactive form [34].

Taken together, the data presented here suggest that autophagy could attenuate the poly (I:C)-induced, TLR3-mediated inflammatory responses in epidermal keratinocytes suggesting autophagy is highly associated with psoriasis, mainly through a cytoprotective mechanism to maintain keratinocyte homeostasis. Further research is needed to better understand the mechanisms of autophagy in immune-mediated skin diseases and to develop new targets for treatments.

## Data Availability

The data used to support the findings of this study are available from the corresponding author upon request.
